# Microstructure, wear, and corrosion properties of PEEK-based composite coating incorporating titania- and copper-doped mesoporous bioactive glass nanoparticles

**DOI:** 10.1039/d4ra07986h

**Published:** 2025-01-21

**Authors:** Khalil Ahmad, Ayman Imran, Badar Minhas, Aqsa Aizaz, Abdul Khaliq, Abdul Wadood, Muhammad Haseeb Nawaz, Muhammad Tajammal Chughtai, Rahila Batul, Muhammad Atiq Ur Rehman

**Affiliations:** a Department of Materials Science and Engineering, Institute of Space Technology Islamabad Pakistan atique1.1@hotmail.com; b Centre of Excellence in Biomaterials and Tissue Engineering, Materials Science Engineering Department, Government College University Lahore 54000 Pakistan; c College of Engineering, University of Hail Saudi Arabia; d College of Pharmacy, University of Hail Saudi Arabia

## Abstract

Poor wear- and corrosion-resistance of 316L SS implants are critical problems in orthopedic implants. This study aims to improve the wear- and corrosion-resistance of 316L SS through surface coating. In this study, a bilayer composite coating consisting of polyether ether ketone (PEEK) as the first layer, and titania (TiO_2_)- and Cu-doped mesoporous bioactive glass nanoparticles (Cu-MBGNs) were deposited as the second layer on a 316L SS *via* electrophoretic deposition (EPD). Scanning electron microscopy (SEM) images of the bilayer composite coating showed the distribution of TiO_2_ and Cu-MBGNs within the PEEK matrix. Energy dispersive spectroscopy (EDS) analysis confirmed the presence of TiO_2_ and Cu-MBGNs in the bilayer composite coating. Fourier transform infrared spectroscopy (FTIR) identified the functional groups attributed to the PEEK, TiO_2_ and Cu-MBGNs. X-ray diffraction (XRD) analysis confirmed the presence of TiO_2_ (anatase) and Cu-MBGNs in the bilayer composite coating. The coating exhibited a strong antibacterial effect against *Staphylococcus aureus* (*S. aureus*) and *Escherichia coli* (*E. coli*). Incorporating TiO_2_/Cu-MBGNs into the bilayer composite coating significantly modified the surface of 316L SS by improving the wear- and corrosion-resistance. Pin on disc test revealed that the specific wear rate of ∼(0.4570 ± 0.009) × 10^−6^ mm^3^ Nm^−1^ of the PEEK coating decreased to (0.0482 ± 0.007) × 10^−6^ mm^3^ Nm^−1^ on incorporating TiO_2_/Cu-MBGNs in PEEK coating under a normal load of 10 N in Dulbecco's Modified Eagle Medium (DMEM). Furthermore, electrochemical impedance spectroscopy (EIS) results revealed that the impedance value of the bilayer composite coating remained ∼4.56 × 10^5^ Ω cm^2^ compared to 8.81 × 10^3^ Ω cm^2^ of 316L SS after 24 h immersion in phosphate-buffered saline (PBS). Thus, this study demonstrated that the wear- and corrosion-resistance of 316L SS can be improved by incorporating TiO_2_/Cu-MBGNs in PEEK-based composite coatings for orthopedic applications.

## Abstract 2

Poor wear and corrosion-resistance of 316L SS implants are critical problems in orthopedic implants.

## Graphical abstract

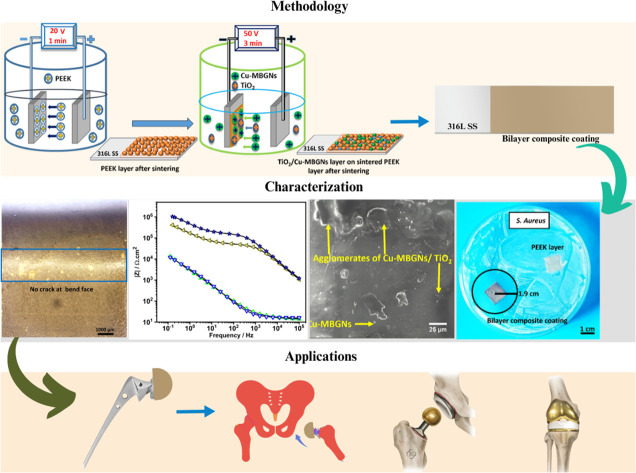

## 1. Introduction

Austenitic low carbon stainless steel (316L SS) is a favourable alloy for load bearing and maintaining structural integrity in orthopedic applications owing to its low cost and ease of fabrication.^1–4^ However, 316L SS corrodes in the human body, which leads to adverse biological reactions^5–7^ due to the uncontrolled release of toxic metal ions (Fe^2+^, Cr^6+^, and Ni^2+^, *etc.*) that cause immunological responses, such as allergies in patients.^8^ Corrosion of 316L SS implants in physiological fluids is a significant cause for failure of the implants, and it is reported that almost 90% of implants fail due to corrosion attack.^9^ Furthermore, the poor wear resistance of 316L SS implants causes loosening of the implant,^10,11^ and wear debris can initiate allergic reactions to surrounding tissues.^12^

The wear and corrosion resistance of 316L SS implants can be improved through surface modification by depositing a biocompatible, bioactive, antibacterial, wear and corrosion-resistance coating on the surface of 316L SS.^13–15^ Bioactive coatings, including 45S5 bioglass (BG), hydroxyapatite (HA), mesoporous bioactive glass nanoparticles (MBGNs), and ceramic coatings, which include alumina and titania, and polymer coatings, such as PEEK coating, are ideal for modifying the surface of 316L SS. These coatings can be deposited *via* electrophoretic deposition technique.^16,17^

EPD is one of the robust techniques for depositing biopolymers, bioceramics, and composite coatings in biomedical implants with simple and intricate shapes at room temperature.^18^ There are two types of EPD, cathodic and anodic, depending on the net charge of the particles acquired in a stable suspension. In the first step of EPD, the particles are charged positively or negatively to form a stable colloidal suspension. An electric field is applied in the second step; the charged particles migrate toward the oppositely charged,^18–20^ and the charged particles are deposited to form a uniform coating.^21^

PEEK is a synthetic thermoplastic that exhibits biocompatibility, wear resistance, chemical resistance, mechanical strength, and radiolucency.^22^ Nevertheless, PEEK as a coating material offers surface modification of metallic implants by coating because the elastic modulus of PEEK (3–4 GPa) is close to that of cortical bone (7–30 GPa).^22,23^ The bio-inertness of PEEK can be mitigated by incorporating bioactive agents.^24^ Cu-MBGNs provide multifunctional activities, such as angiogenesis (release of Cu^2+^ ions), osteogenesis, and osteopromotive release of Ca and P ions, and an antibacterial effect inhibiting biofilm formation.^25–27^ Titania (TiO_2_, anatase phase) is a highly biocompatible, wear- and corrosion-resistant ceramic material recommended for orthopedic applications, such as artificial hip and knee replacements. When TiO_2_ is used for bone tissue replacement, it enhances the implant's integration with host tissues.

Various researchers have recently studied PEEK-based coatings.^28,29^ These coatings focus on achieving bioactive and antibacterial properties.^30,31^ However, the mechanical properties are yet to be investigated in detail. Thus, this is a critical research gap. Poor adhesion between the two layers deposited *via* EPD can result in the release of wear debris. Resistance improving the coating's adhesion strength, corrosion resistance, and wear resistance is essential.^32–34^ Rehman *et al.*^35^ deposited a PEEK/BG layer on 316L SS to improve wear resistance, corrosion resistance, and bioactivity. The chitosan/Lawson/BG was deposited on the PEEK/BG layer to impart the antibacterial property and to provide the controlled release of Lawson. Nawaz *et al.*^31^ developed chitosan/gelatin/Ag–Mn MBGN composite coating on the PEEK/BG layer (deposited on stainless steel *via* EPD) to upregulate osteoblast cell attachment and proliferation in addition to antibacterial activity. Rehman *et al.*^36^ reported that PEEK and PEEK/bioactive glass composite coatings could withstand a 7 N applied load during a tribology test. Kumari *et al.*^37^ incorporated an extract of jack fruit latex resin into poly(methyl methacrylate) (PMMA) to modify the antibacterial and bioactive properties of dental-based materials. Kumari *et al.*^38^ also developed PMMA-based zirconia reinforced bio nanocomposites to improve the structural, mechanical and biological properties of dental applications. Kumari *et al.*^39^ developed PMMA-based nanocomposite materials by incorporating MgO for dental applications.

Previous studies followed a “sintered-then-deposit” sequence for PEEK-based composite coatings, while in this study, we innovatively explored a deposit-then-sintered approach. This newly developed process improved the wear- and corrosion-resistance of 316L SS implants for orthopedic applications. In previous studies on PEEK-based composite coatings, the PEEK layer was first deposited and sintered. Afterward, the second layer of bioactive glass particles was deposited.

The primary purpose of this study is to address the limitations of the adhesion strength of PEEK coating in tribological analysis. In our previous study,^40^ a PEEK coating was delaminated under a 1 N normal applied load and subjected to a pin-on-disc test in the presence of Dulbecco's Modified Eagle Medium (DMEM), and its adhesion strength was compromised. The present study investigated the synergistic effect on the final coating surface developed by incorporating TiO_2_/Cu-MBGNs in a PEEK coating deposited on 316L SS electrophoretically and sintered to improve wear- and corrosion-resistance. This study aims to improve the wear- and corrosion-resistance of 316L SS in human physiological fluid. The information obtained from this study can further be helpful for *in vivo* trials and clinical investigations, leading to commercialization. An improvement in the wear- and corrosion-resistance of the 316L SS substrate will enhance the lifespan of orthopedic implants, reduce the risk of implant failure, and improve patient satisfaction.

The significance of this study demonstrates that incorporating the TiO_2_/Cu-MBGNs in bilayer composite coating improves the structural morphology by eliminating the micro-voids in the PEEK layer. This improved morphology of the bilayer composite coating ultimately unprecedently improved the wear- and corrosion-resistance of the bilayer composite coating. This bilayer composite coating has significant potential for orthopedic applications.

## 2. Materials and methods

### 2.1. Materials

AISI 316 L SS foil of 1 mm thickness was used to prepare the substrates for coatings. The composition of 316L SS was Cr-16.5%, Ni-10%, Mo-2%, N-0.10%, S-0.015%, P-0.045%, C-0.03%, Si-1.00%, Mn-2.00%, and Fe (balance%).^41^ PEEK 704 was purchased from Victrex, UK. TiO_2_ powder (CAS-No. XF 180, purity ≥99.5%) was purchased from XFNANO, China. The chemicals (hexadecyltrimethyl ammonium bromide (CTAB, CAS-No. 57-09-0, purity ≥99%), calcium nitrate tetrahydrate (CAS-No. 13477-34-4, purity ≥99.0%), ammonia solution (CAS-No. 1336-21-6, purity = 32%), and ethyl acetate (CAS-No. 141-78-6, purity ≥99.5%)) for the synthesis of Cu-doped MBGNs were purchased from Sigma-Aldrich (Germany). Tetraethyl orthosilicate (TEOS, CAS-No. 78-10-4, purity ≥99.0%) was purchased from Merck Millipore (Germany).

### 2.2. Methods

A sheet cutter was used to cut a 316L SS sheet in the dimension of (3 × 1.5) cm^2^. The substrates were washed and cleaned with a solution of acetone, followed by washing with ethanol, keeping their ratio as 1 : 1 in an ultra-sonication bath for 10 min. Then, the substrates were rinsed in distilled water and dried in air.

Copper-doped mesoporous bioactive glass nanoparticles (Cu-MBGNs) with a composition of (70 mol% SiO_2_, 25 mol% CaO, and 5 mol% CuO)^26^ were synthesized by applying the modified Stöber method.^42^ First, 2.24 g of C-TAB (soft template) was dissolved in 104 mL of water, and continuous stirring was performed for 15 min. Then, 32 mL of ethyl acetate was added dropwise to the solution. Afterward, 22 mL of ammonium hydroxide (28%) was used to maintain a pH ∼9–10, and then 23.04 mL of TEOS was added dropwise in the solution *via* burette. Finally, 5.21 g of calcium nitrate and copper nitrate were added to the solution. Copper nitrate was added according to the desired composition, which is 5 mol% – (0.869 g in 5.21 g). The solution was stirred at 250 rpm for 4 h and then placed at room temperature for 12 h. Then, the suspension was centrifuged at 10 000 rpm for 10 min from the parent's solvent and washed four times with ethanol and distilled water equally. These particles were dried at 37 °C for 24 h and then calcined at 700 °C for 3 h at a 10 °C min^−1^ heating rate in the box furnace (KSL-1700X-A7), and then particles were furnace cooled and used in the development of TiO_2_/Cu-MBGNs layer.^43^

In this study, the PEEK coating was deposited as the first layer (base layer) on 316L SS *via* EPD with an applied electric field of 20 V cm^−1^ with less deposition time, *i.e.*, 1 min instead of 3 min, to achieve a relatively thin coating.^40^ A stable suspension containing 20 g L^−1^ PEEK (∼10 μm average particle size) in 50 mL of chitosan solution (chitosan solution of 0.5 g L^−1^ was prepared separately) was prepared.^44,45^ Chitosan solution was used to prepare the suspension owing to its suitability for the deposition of bioceramics, as discussed in previous studies.^14,46^ The suspension of PEEK was subjected to magnetic stirring for 30 min, followed by ultrasonication for 30 min and magnetic stirring for 10 min. The pH of the PEEK suspension was maintained between 4 and 5 because, in this pH value, the PEEK particles, along with chitosan macromolecules, were positively charged in the suspension. A detailed investigation of the optimization of EPD parameters along with deposition mechanisms was presented in ref. 40. When the stable PEEK suspension was subjected to EPD, the positively charged PEEK particles were deposited on the oppositely charged electrode of 316L SS (cathode) under the influence of a 20 V cm^−1^ applied electric field with a deposition time of 1 min. The reactions involved in the deposition mechanism of PEEK are illustrated in Subsection 3.1. After deposition of the PEEK coating on 316L SS, the coatings were subjected to sintering at 350 °C/30 min at a heating rate of 10 °C min^−1^ for complete densification.^40^

**Fig. 1:**
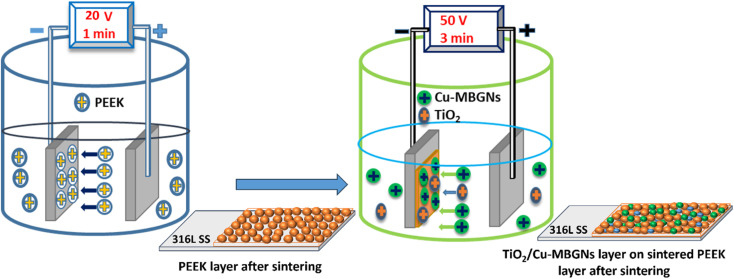
Schematic of deposition and sintering steps of the PEEK layer and TiO_2_/Cu-MBGN layer on 316L SS *via* EPD.

The second layer (the top layer) of TiO_2_/Cu-MBGNs was deposited on the sintered PEEK layer. A stable suspension of TiO_2_/Cu-MBGNs was prepared in the ethanol solution, in which 0.1 g of TiO_2_ (anatase) (0.2% w/v) and 0.2 g of Cu-MBGNs (0.4% w/v) were added to a 50 mL ethanol solution to prepare a stable suspension. A 0.1 g of TiO_2_ was added to 0.2 g of Cu-MBGN suspension to improve mechanical, tribological, and corrosion resistance properties.^47,48^ A systematic study was conducted to optimize the required concentration of TiO_2_ in the Cu-MBGN suspension to obtain the desired properties. First, TiO_2_ was added at a minimum concentration, *i.e.*, 0.025 g in Cu-MBGN suspension in ethanol, which improved some properties. Moreover, it could not impart the desired mechanical (adhesion strength), tribological, and corrosion resistance levels. Thus, the concentration of TiO_2_ was increased with an interval of 0.025 g in 0.2 g in a 50 mL suspension of Cu-MBGNs. The desired properties were achieved at a concentration of 0.1 g TiO_2_ in 0.2 g Cu-MBGNs in a 50 mL suspension. The suspension of TiO_2_/Cu-MBGNs in ethanol solution was subjected to magnetic stirring for 1 h, followed by probe sonication for 40 min. This process was repeated three times until the particles were homogenized in the suspension. Furthermore, the pH of the suspension was maintained at 3–4 by adding 3 mL of acetic acid dropwise in the suspension. In this pH range, particles of TiO_2_ and Cu-MBGNs were positively charged in the ethanol suspension. The PEEK coating, after sintering, was immersed in a stable suspension of TiO_2_/Cu-MBGNs. An electric field of 50 V cm^−1^ for 3 min was applied to the suspension. The positively charged particles of TiO_2_ and Cu-MBGNs were forced to move and deposit onto the sintered PEEK layer already deposited on the 316L SS electrode (cathode), producing a uniform coating (second layer) on 316L SS.^48,49^ The second layer of TiO_2_/Cu-MBGNs was deposited at a higher applied electric field and deposition time because a higher driving force was required to overcome the nonconductive sintered PEEK layer already deposited on 316L SS. After the deposition of the second layer, the coated samples were dried at room temperature and then again sintered at 350 °C in the box furnace for 30 min at a 10 °C min^−1^ heating rate, followed by furnace cooling.^33,48^ The newly developed sintering cycle densified and compacted both layers into a single layer. Moreover, deposition was made into layers, so the developed coating was called a bilayer composite coating. The deposition mechanism of TiO_2_/Cu-MBGNs on the sintered PEEK layer is illustrated in Subsection 3.1. Fig. 1 shows the sequential steps of deposition and sintering of the PEEK layer and TiO_2_/Cu-MBGNs layer on 316L SS *via* EPD.

The sintering temperature was selected because of the trial-error approach. The bilayer composite coating samples were subjected to various characterization techniques, including scanning electron microscopy (SEM/EDS); Fourier transform infrared spectroscopy (FTIR); surface properties in terms of surface roughness and contact angle; adhesion strength; biological studies in terms of antibacterial activity and cellular study; and wear and corrosion analysis.

### 2.3. Characterization of bilayer composite coating

#### 2.3.1. Morphological and cross-sectional analysis

SEM (AURIGA 4750, Carl Zeiss AG, Germany) was used to study the surface morphology and cross-sectional analysis of the bilayer composite coating. The accelerating voltage during the morphological analysis was 10 kV with a working distance of 50 mm. A cross-section of the bilayer composite coating was prepared by grinding it on sandpaper (grit size 600), and all dirt particles were removed using a blower at the edge. The bilayer composite coating samples were sputtered with conductive materials such as gold/palladium (Au/Pd) on the coating surface by (Q150/S Sputter) to avoid the charging effect during analysis. The (Au/Pd) sputtering thickness was ∼5 nm.^50^ In addition to morphology and cross-sectional analysis, elemental mapping of the coating was carried out by energy dispersive spectroscopy (EDS), which was performed in conjunction with SEM to depict the existence of different elements and their spatial distribution on the surface of the bilayer composite coating.

#### 2.3.2. Identification of functional groups

FTIR spectroscopy (Nicolet 6700 Summit lite Thermal Scientific) analyzed the presence of functional groups of TiO_2_, Cu-MBGNs, sintered PEEK coating, and bilayer composite coating and their shifting at a particular wavenumber (cm^−1^). The transmittance spectra were recorded from 2000–400 cm^−1^ wavenumber with 40 scans per spectrum and a resolution of 4 cm^−1^.

#### 2.3.3. Thermogravimetric analysis

The thermal stability of bilayer composite coatings was determined using a TGA/DSC 1 STAR® analyzer from METTLER TOLEDO. Thermal analysis was carried out by recording the weight changes by increasing the temperature to 900 °C. Weight changes may occur due to evaporation, phase change, and decomposition processes. This analysis used 10 mg of bilayer composite coating powder. The measurements were carried out in an alumina crucible, and nitrogen gas provided an inert environment. The blank test was performed using a blank alumina crucible without a sample for accurate results under the 25–900 °C dynamic conditions and a heating rate of 10 °C min^−1^. Sample measurement was performed after the blank test following similar dynamic conditions. The char yield (Yc) and degradation temperatures for various steps were also recorded.

#### 2.3.4. XRD analysis

X-ray diffraction (XRD: Panalytical diffractometer-PW3719) analysis was used to identify the phases in the bilayer composite coating deposited on 316L SS *via* EPD. A uniform coating was developed on 316L SS to avoid diffraction artifacts during the analysis. The sample was sintered to ensure crystallization and polished for a smooth surface. The XRD setup employed a Cu anode (Cu Kα radiations with a wavelength of 1.540598 Å). Other parameters were the tube voltage of 40 kV and the tube current of 30 mA with a step size of 0.05° 2*θ*. The 2*θ* angle was scanned from 15° to 80° for bilayer composite coating powder to obtain all the relevant peaks associated with TiO_2_ and Cu-MBGNs.^47^

#### 2.3.5. Surface properties

The values of the average surface roughness (*R*_a_) of the bilayer composite coating and 316L SS were measured by applying a stylus profilometer (TMR 360). A diamond-tipped stylus underwent a reciprocating motion of about ∼5 mm on bilayer composite coating and 316L SS. The stylus movement was recorded as a signal, and an inbuilt software in the profilometer processed this data and calculated the values of *R*_a_ of the bilayer composite coating and 316L SS (where *n* = 5 and mean values were reported along with the standard deviation). The wettability in terms of contact angle was measured manually using the Sessile drop method by dispensing a 5 μL drop of distilled water on surfaces of 316L SS and bilayer composite coating. Image J software was used to measure the contact angle between the water drop and the bilayer composite coating. The mean values (where *n* = 5) of the contact angle measurements were reported along with the standard deviation. The contact angle of the surface determines the cell adhesion, cell attachment, and proliferation of cells and proteins on the implant surface; the hydrophilic or hydrophobic properties of the surface were determined.

#### 2.3.6. Bend test (deformability)

Deformability is an essential characteristic of bilayer composite coating. It was evaluated through a bend test according to the ASTM B571-97 standard. Forceps bent the bilayer composite-coated sample at an angle of 180°. Afterward, a stereomicroscope (Nikon SMZ 25, Japan) was used to take images at the bend face and edge face to observe any sign of cracking or delamination of the bilayer composite coating from 316L SS.

#### 2.3.7. Antibacterial study

Biological studies were conducted regarding antibacterial and alkaline phosphate (ALP) activity. The antibacterial study was carried out to investigate the antimicrobial effect of Cu-MBGNs against Gram-positive *Staphylococcus aureus* (*S. aureus*) and Gram-negative *Escherichia coli* (*E. coli*) by disk diffusion test, also known as (Kirby–Bauer) test. The PEEK layer and bilayer composite coating were subjected to disc diffusion tests to determine the antimicrobial effect of Cu-MBGNs. An agar medium was subsequently prepared using nutrient agar (Oxoid-UK) and distilled water, autoclaved for 20 min at 121 ± 1 °C, and 20 mL of the conditioned medium was poured into sterile Petri dishes and solidified at room temperature. Later, 10 μL of *S. aureus* and *E. coli* with an optical density (OD_600_) of 0.015 ± 0.02 were uniformly inoculated onto the agar plates. The bilayer composite coating samples were exposed to UV light for 2–3 h to avoid contamination. Subsequently, the PEEK layer and bilayer composite coating samples were placed over inoculated agar and incubated at 37 °C for 24 h. After that, the images were captured, and the inhibition zones were measured using Image J software. The disk diffusion test was repeated three times against each bacterial strain, and the mean values and standard deviations were reported.

#### 2.3.8. Cellular study

The human ALP activity of human fetal osteoblastic (hFOB) 1.19 cells co-cultured with 316L SS and bilayer composite coating was evaluated by the human ALP ELISA kit (Wuhan Fine Biotech Co., Ltd) per the manufacturer's protocol. Briefly, after a 14 days culture, the culture medium was removed and washed three times with pre-cooling PBS, followed by the addition of cell lysate and protease inhibitor. The osteoblastic cells were transferred to mini-centrifuge tubes and incubated on ice with interval ultrasonic shocks for membrane disruption and to avoid sticky DNA. Later, the tubes were centrifuged at 10 000 rpm for 10–12 min at 2–8 °C, and the supernatant was transferred to the well plate with coated capture antibody (Ab), followed by static incubation for 90 min. Similarly, the standard given was also diluted in different concentrations and added to the wells. The reagents, such as a biotin-labelled antibody, mean specific antibody binding capacity (SABC), and tumour mutational burden (TMB), were added and incubated per the manufacturer's instruction, followed by absorbance measurements at a wavelength of 450 nm. The concentration of ALP release from hFOB 1.19 cells co-cultured with uncoated and coated cells was calculated by comparing it with the standard curve.^51^

#### 2.3.9. Tribological study

A pin-on-disk test, which is a fundamental test of tribology (Tribometer (MT/60/NI, Spain) to determine the friction and wear behavior of PEEK coating of a thickness (66 μm) in a previous study, was performed under dry condition.^40^ In this study, PEEK coating and bilayer composite coating deposited on 316L SS were subjected to a pin-on-disk test under wet conditions in the presence of Dulbecco's Modified Eagle Medium (DMEM). A diamond indenter with a speed of 50 rpm was used for the pin-on-disc test of PEEK and bilayer composite coatings under an applied load of 10 N with a 30 m sliding distance in the presence of DMEM (wet condition at 28 ± 3 °C and humidity level of 63 ± 5%) to mimic the human body environment. A coefficient of friction (CoF) is a function of sliding distance, cumulative wear volume, and specific wear rates of PEEK and bilayer composite coatings. The specific wear rates of PEEK and bilayer composite coatings were compared to observe the combined effect of TiO_2_ and Cu-MBGNs in the bilayer composite coating. The specific wear rates of the PEEK coating and the coating in DMEM were calculated using eqn (1):1
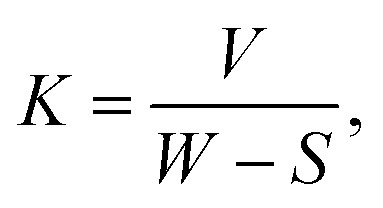
where *V* = cumulative wear volume (mm^3^), *W* = applied load (N), and *S* = sliding distance (m) and *K* = specific wear rate (mm^3^ Nm^−1^).

#### 2.3.10. Electrochemical studies

The electrochemical impedance spectroscopy (EIS) technique was used to analyse the electrochemical response of the 316L SS and bilayer composite coating. The corrosion behavior of 316L SS and bilayer composite coating was investigated in PBS at 37 ± 1 °C to mimic the human body fluid environment. These samples were immersed in PBS, and analysis was performed after 1 h and 24 h to assess the corrosion behavior of the bare 316L SS and coating. The electrochemical response of these samples was also investigated in simulated body fluid (SBF) to analyse corrosion resistance. A typical three-electrode system uses 316L SS and bilayer composite coating substrates as a working electrode, graphite electrode as a counter electrode, and Ag/AgCl as a reference electrode. The substrates of 316L SS and the coating were sealed with epoxy resin, leaving an area of 1 cm^2^. The EIS measurements were carried out with a potentiostat/galvanostat (Gamry Instrument, ref. 600, England). EIS tests were conducted in the 10^−1^ to 10^5^ Hz frequency range with 10 mV perturbation. Each test was recorded three times to ensure the reproducibility of the results.

## 3. Results and discussion

### 3.1. Mechanism of EPD

The layers of the PEEK and TiO_2_/Cu-MBGNs were deposited on 316L SS *via* EPD. However, a 0.5 g L^−1^ chitosan solution was used as a binder to deposit these coatings. The existing literature reported the deposition of chitosan with ceramic particles (hydroxyapatite, alumina, titania, metallic ion-doped bioactive glass, and bioactive glass) as co-deposition on 316L SS *via* EPD.^36,47–49,52^ Chitosan is insoluble in water, but it is soluble in a solution of distilled water, acetic acid, and ethanol, as reported in ref. 14 and 53 because the amine groups of chitosan are protonated (become positively charged) at low pH ∼4–5. The dissociation reaction of acetic acid and protonation reaction of chitosan according to reactions (2) and (3):

2 CH_3_COOH + H_2_O → CH_3_COO^−^ + H_3_O^+^

3 Chitosan–NH_2_ + H_3_O → Chitosan–NH_3_^+^ + H_2_O

These positively charged macromolecules of chitosan were forced to move toward the cathode as the potential was applied,^18,54^ but the reduction reactions of water and oxygen occurred at the cathode by reactions (4) and (5):^55,56^

4 2H_2_O + 2e^−^ → H_2_ + 2OH^−^

5 O_2_ + 2H_2_O + 4e^−^ → 4OH^−^

As reactions (4) and (5) occurred at the cathode, the pH increased, and positively charged macromolecules of chitosan lost their charge and were deposited on the cathode as an insoluble material according to reaction (6):

6 Chitosan–NH_3_^+^ + OH^−^ → Chitosan–NH_2_ + H_2_O

Because previous studies have demonstrated that chitosan can be used to deposit inorganic materials on metallic substrates as co-deposition,^57–60^ the positively charged chitosan macromolecules were used to deposit the PEEK particles when the PEEK suspension was prepared in the 0.5 g L^−1^ chitosan solution by magnetic stirring and ultrasonication. These positively charged macromolecules of chitosan are adsorbed on the surface of PEEK particles by imparting a net positive charge.^58^ These positively charged PEEK particles were deposited on the cathode (316L SS) when 20 V with a deposition time of 1 min was applied. Eqn (7) shows the proposed reaction of chitosan and peek particles (co-deposition) on 316L SS. The interaction between the positively charged macromolecules of chitosan and PEEK particles was physical.7
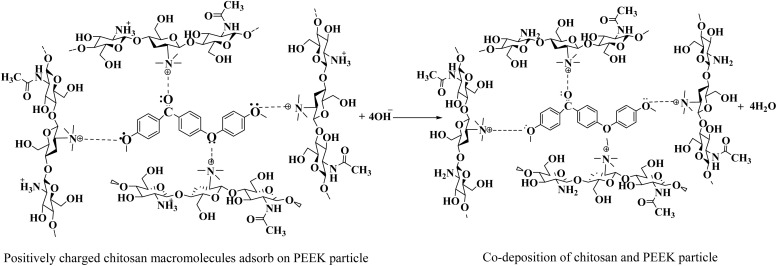


After PEEK particle deposition, the coating was subjected to sintering at 350 °C for 30 min at a 10 °C min^−1^ heating rate. After the sintering process, the chitosan was evaporated, and only PEEK particles were deposited on 316L SS. The proposed reaction of PEEK particles surrounded by chitosan macromolecules after sintering at 350 °C for 30 min is shown in eqn (8):8
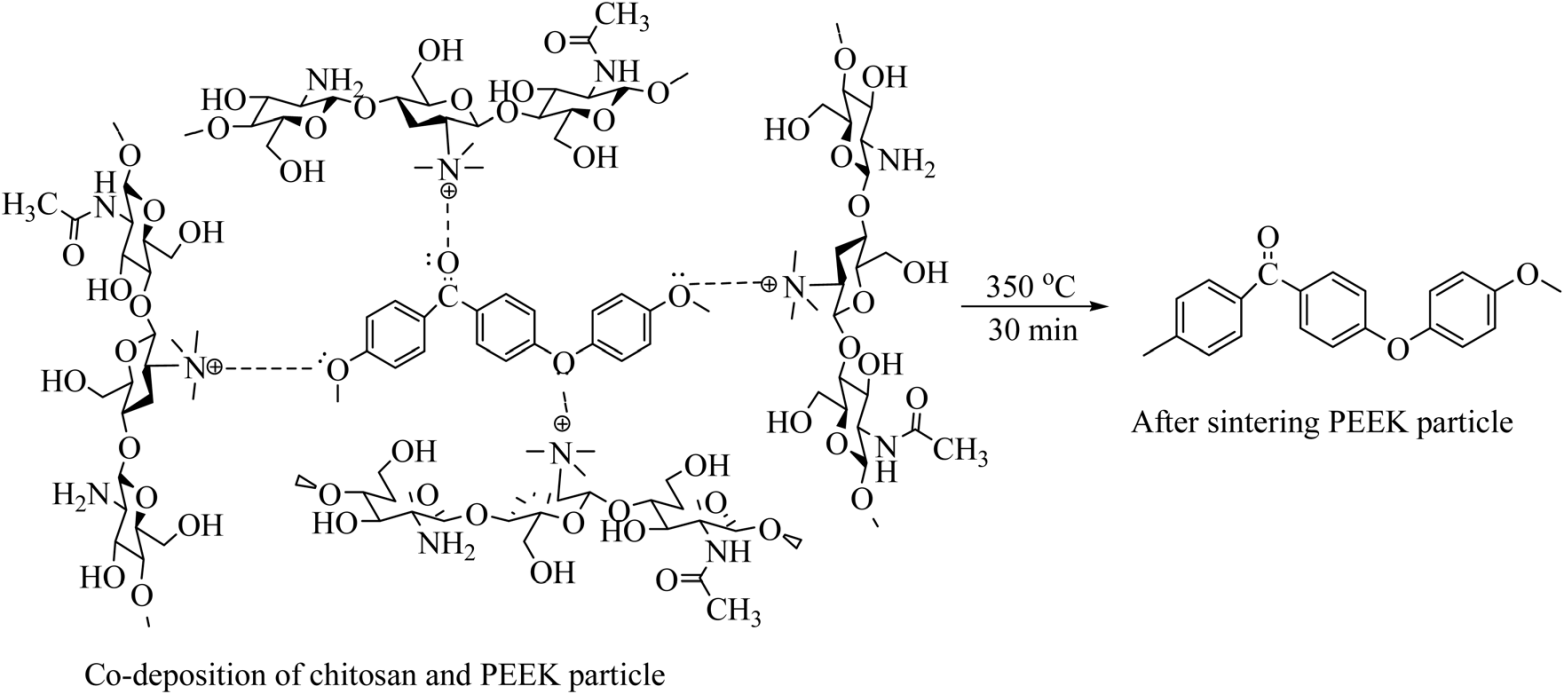


Cu-MBGN particles were positively charged in the ethanol solution and were deposited on the cathode (316L SS) along with TiO_2_ particles.^49,61^ In the TiO_2_/Cu-MBGN suspension in ethanol, acetic acid was added to adjust the pH of the suspension. At pH ranging from ∼3 to 4 of the ethanol and acetic acid suspension, the protonation reactions occurred between the acetic acid and Cu-MBGNs because the silanol group (–Si–OH) at the surface of the Cu-MBGNs was protonated based on reactions (9) and (10):^49,62,63^

9 –Si–O–Cu^2+^ + CH_3_COO^−^ + H^+^ → –Si–OH–Cu^2+^ + CH_3_COO^−^

10 –Si–OH–Cu^2+^ + H^+^ → –Si–OH_2_^+^–Cu^2+^

The protonated silanol group (–Si–OH_2_^+^–Cu^2+^) was forced to move toward the cathode to deposit when a potential of 50 V with a 3 min deposition time was applied to the TiO_2_/Cu-MBGN suspension. It can be assumed that when the protonated silanol group reaches the cathode surface, oxygen or water reduction reactions (4) and (5) might produce hydrogen gas or hydroxyl ions. This protonated silanol group lost its positive charge and was deposited as a neutral silanol group (Si–OH) on 316L SS.^64^ Similarly, as in the suspension of TiO_2_/Cu-MBGNs, TiO_2_ particles were also deposited with Cu-MBGNs on sintered PEEK coating at 50 V with a deposition time of 3 min. TiO_2_ particles were also positively charged in the ethanol and acetic acid suspension, with pH ranging from ∼3 to 4. A previous study also reported that TiO_2_ particles were positively charged in ethanol and acetic-based suspension and deposited at ∼50 V with 3 min of deposition time.^48^

### 3.2. SEM analysis of PEEK coating and bilayer composite coating

**Fig. 2:**
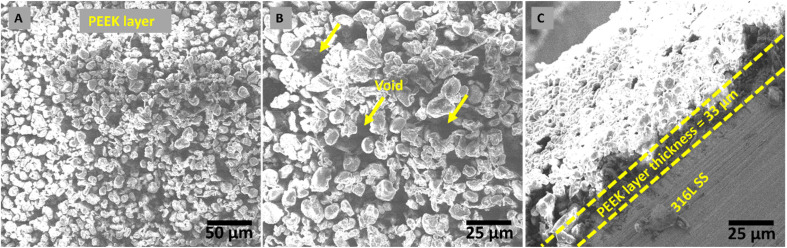
Morphology of a PEEK layer deposited at 20 V and 1 min, followed by sintering at 350 °C. (A) Shows the morphology of PEEK layer deposited on 316L SS *via* EPD, (B) shows the voids present in PEEK layer, and (C) shows the thickness of PEEK layer.

**Fig. 3:**
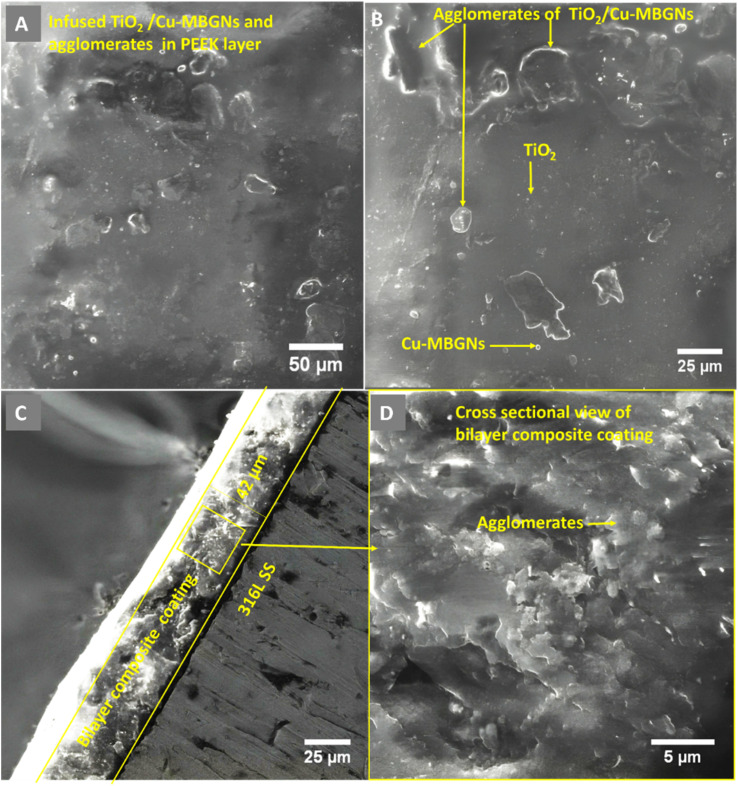
SEM images of the coating produced at 50 V, 3 min: (A) low magnification showing the deposition of coating; (B) high magnification showing the distribution of TiO_2_/Cu-MBGNs particles on the PEEK layer; (C) cross-section showing coating thickness; and (D) morphology of cross-section of bilayer composite coating.

The morphological analysis of the PEEK layer was deposited at 20 V with a deposition time of 1 min on 316L SS and then sintered at 350 °C for 30 min. A bilayer composite coating was deposited at 50 V with a deposition time of 3 min on a sintered PEEK coating. SEM images of the PEEK layer and bilayer composite coating are shown in Fig. 2 and 3, respectively. The SEM image in Fig. 2A PEEK layer at a magnification of 500× shows the uniform morphology of the PEEK layer all over the surface of the 316L SS substrate without any discernible cracks. However, numerous micro voids were observed in the morphology of the PEEK layer at closer observation of Fig. 2B at a magnification of 1000×. The overall morphology of the PEEK layer is also consistent with the schematic diagram (Fig. 1). However, Fig. 2C at a magnification of 1000× shows that the thickness of the PEEK layer is ∼33 μm.^65^ The morphological results of the PEEK layer are consistent with earlier findings reported by Corni *et al.*^65^

Fig. 3 shows the SEM images (A and B) at a magnification of 1000× of bilayer composite coating, with smooth and compacted morphology without any discernible pores, micro voids, or cracks in the bilayer composite coating compared to the PEEK layer (Fig. 2). Fig. 3B at 1000× also shows the preferential adhesion and agglomerates/clustering of TiO_2_ and Cu-MBGNs at specific areas of the bilayer composite coating, which can be attributed to differences in wettability or interfacial interaction between the PEEK layer and TiO_2_/Cu-MBGN particles. Baştan *et al.*^52^ reported a morphology similar to the co-deposition of PEEK/HA composite coating. Fig. 1 schematically shows the deposition mechanisms (3.1 Mechanism of EPD) of both layers and their purposed reactions (2–10) involved in deposition, which constituted the bilayer composite coating. Furthermore, the SEM images (Fig. 3A and B) of the bilayer composite coating revealed a remarkable improvement in the microstructure of the bilayer composite coating by depositing the TiO_2_/Cu-MBGNs layer on the PEEK layer.

Fig. 3C at a magnification of (1250×) showed the image at the cross-section of the bilayer composite coating, which revealed a coating thickness of ∼42 μm (deposition parameters: 50 V cm^−1^ and 3 min), while the previous study of the co-deposition of PEEK/HA coating exhibited ∼70 μm.^52^ The difference in coating thickness between bilayer composite coating and co-deposition was significant because, after deposition of the TiO_2_/Cu-MBGN layer on the sintered PEEK layer, both layers were sintered at the same parameters, resulting in compacting both layers and reduced coating thickness, which was lower than previous studies reported.^32,35,49,66,67^

Fig. 3D at (2000×) shows the high magnification at the cross-sectional morphology of the bilayer composite coating, which demonstrates that the coating is densely packed. A similar coating thickness for the PEEK-based composite coating was reported in ref. 44 and 52. It is important to note that the interface between the two layers was not distinguished. This could be because the maximum roughness of the PEEK coating (first layer) is ∼10 μm. Thus, the top layer usually infiltrates the bottom layer. Therefore, a clear interface for a PEEK-based multilayer coating cannot be observed. A similar effect was reported in ref. 28, 29 and 31.

Furthermore, it was hypothesized that upon sintering both layers, the bottom PEEK layer melted (because the melting point of PEEK is 343 °C). Thus, the TiO_2_/Cu-MBGNs on the top layer were wetted by the melted PEEK layer, and upon cooling, the TiO_2_ and Cu-MBGNs became trapped in the PEEK layer. These particles acted as reinforcements in the PEEK matrix, thus resulting in excellent adhesion strength. Furthermore, excellent compaction/densification of both layers upon sintering resulted in improved wear- and corrosion-resistance. Similar findings about the strengthening mechanism of PEEK-based composite coatings deposited on 316L SS were reported in previous studies.^32,49,66,68^

### 3.3. EDS analysis

**Fig. 4:**
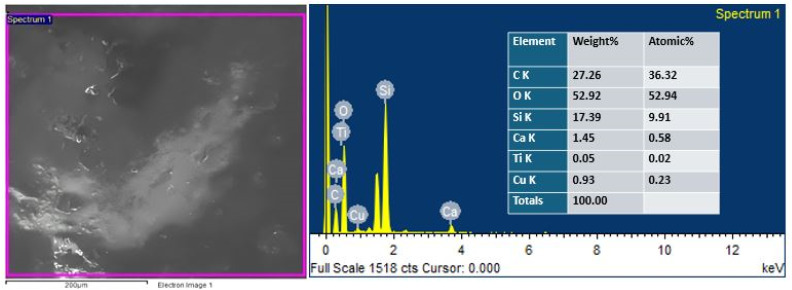
EDS analysis of the bilayer composite coating deposited on 316L SS.

EDS analysis was performed by SEM equipped with an EDS detector to identify and confirm the presence of TiO_2_ and Cu-MBGNs in the bilayer composite coating. The EDS results revealed that carbon and silicon belong to PEEK and that Cu-MBGNs are present as primary constituents. Meanwhile, Ca, Ti, and Cu were present in minor amounts because their concentration in the bilayer composite coating was low. Fig. 4 shows the EDS analysis of the bilayer composite coating.

**Fig. 5:**
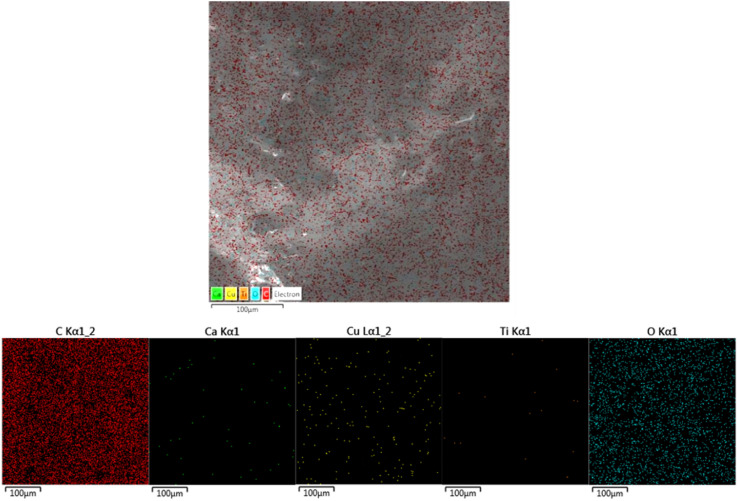
EDS elemental mapping images, which confirms the presence of TiO_2_/Cu-MBGNs in the bilayer composite coating.

Fig. 5 shows the EDS elemental mapping of the bilayer composite coating, which also confirms the presence of TiO_2_ (Ti detected in EDS mapping) and Cu-MBGNs (Si, Ca, and Cu elements indicate the presence of Cu-MBGNs) infused in the PEEK layer.^49^

### 3.4. Identification of functional groups

**Fig. 6:**
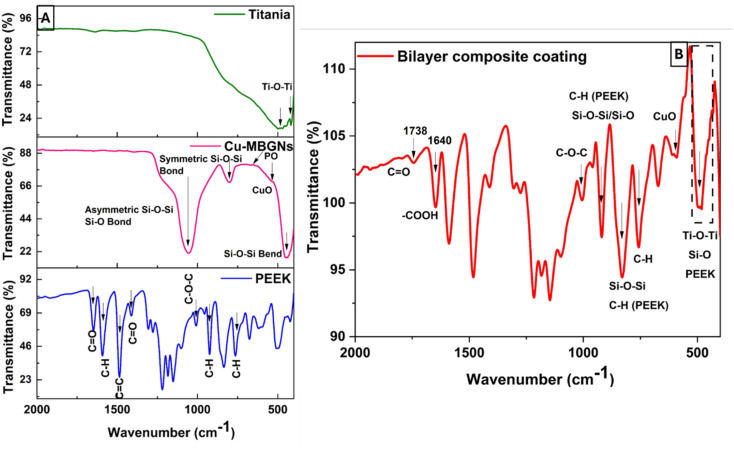
FTIR spectra of (A) PEEK, Cu-MBGNs, TiO_2_, and (B) bilayer composite coating.

**Fig. 7:**
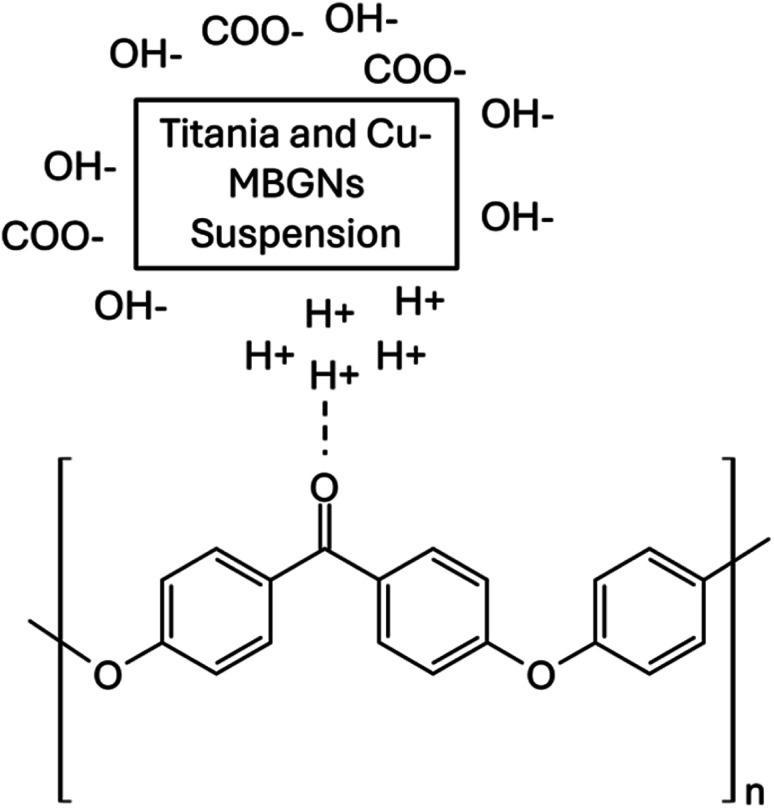
Possible interactions between the sintered PEEK layer and TiO_2_/Cu-MBGN layer.

The ATR-FTIR spectra of PEEK, Cu-MBGNs, and TiO_2_ are presented in Fig. 6A, while the spectrum of the bilayer composite coating is shown in Fig. 6B. The characteristic bands of aromatic rings (C–H) in PEEK were identified at 762, 834, and 924 cm^−1^.^52^ The typical carbonyl (C=O) stretching band and C–O–C in PEEK were detected at 1648 and 1006 cm^−1^, respectively.^61^ Furthermore, a band associated with the angular deformation of the C–H bond was visible at 1590 cm^−1^.^69^ The FTIR spectrum of the Cu-MBGNs showed two bands at 440 cm^−1^ and 812 cm^−1^, which are attributed to Si–O–Si bending and symmetric stretching vibrations, respectively. The bands of asymmetric Si–O–Si (bridging bonds) and Si–O (non-bridging bonds) vibrations were observed in the range of 1299–900 cm^−1^.^26,70^ The appearance of these bands shows that tetrahedrons are formed to create a glass network of particles. The band associated with the phosphate group of MBGNs appeared at 600 cm^−1^.^71^ The band at 545 cm^−1^ corresponded to the presence of Cu–O in Cu-MBGNs.^26^ The presence of TiO_2_ in the composite coatings was illustrated by the characteristic band at 418 cm^−1^ and 482 cm^−1^, which is related to the bending and stretching modes of Ti–O–Ti, respectively.^72^ ATR-FTIR spectroscopy also revealed characteristic bands corresponding to the functional groups of the bilayer composite coating. The formation of a layer with a suspension containing TiO_2_ and Cu-MBGNs over the PEEK-coated substrate was confirmed by FTIR. The carbonyl group of PEEK particles interacted with the hydrogen ions of suspensions containing water, ethanol, and acetic acid, forming weak dipole–dipole interactions. The appearance of a new band at 1738 cm^−1^ corresponded to the aldehyde carbonyl group involved in forming interactions between bilayers,^73^ as shown in Fig. 6B. This can further be confirmed by the broadening and increased intensity of Ti–O–Ti, Cu–O (shifted to 595 cm^−1^), and Si–O–Si symmetric stretching vibration bands. Additionally, the slight shift in the band position of C–O–C confirmed the formation of a bilayer composite coating. The bands at 812 and 595 cm^−1^ were attributed to Si–O–Si symmetric stretching vibrations and Cu–O, respectively, of Cu-MBGNs.^26,70^ The appearance of a broadened and increased intensity band at 482 cm^−1^ corresponded to Ti–O–Ti, Si–O, and PEEK.^26,70,72^ The typical carbonyl (C=O) stretching band and C–O–C in PEEK were detected at 1648 and 1006 cm^−1^, respectively.^61^Table 1 summarises all bands in PEEK, Cu-MBGNs, TiO_2_, and coating deposited on 316L SS *via* EPD. The possible interaction after sintering at 350 °C between the sintered PEEK layer and TiO_2_/Cu-MBGNs layer is shown in Fig. 7.

**Table 1:** Characteristic bands of PEEK, Cu-MBGNs, TiO_2_ and bilayer composite coating

Wave number (cm^−1^)	Corresponding bonds	Associated material	Reference
762, 834, 924	C–H aromatic rings	PEEK	69
1590	C–H angular deformation	PEEK	69
1648, 1738	C=O	PEEK, bilayer	61 and 73
440	Si–O–Si	MBGNs	70
812	NBO	MBGNs	26
418, 482	Ti–O–Ti	TiO_2_	72

### 3.5. Thermogravimetric analysis

**Fig. 8:**
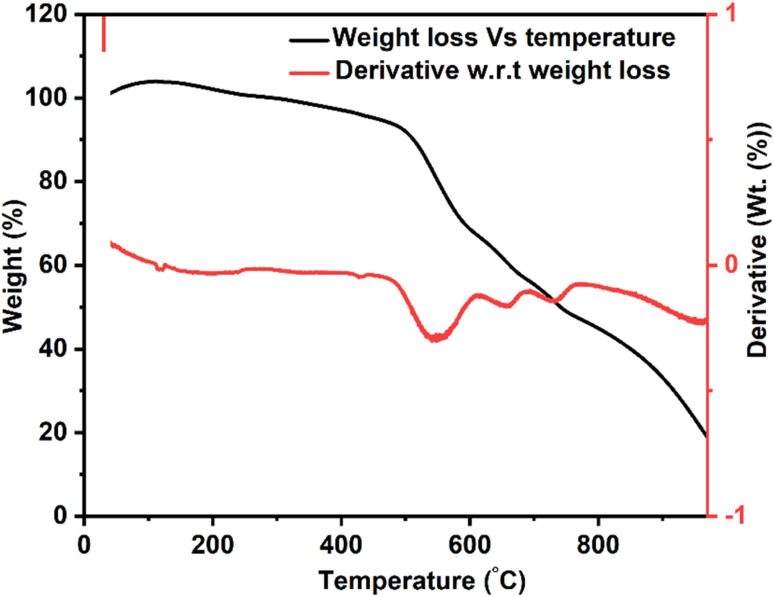
TGA thermogram for bilayer composite coating.

The TGA thermogram of the bilayer composite coating showed three distinct degradation steps, as shown in Fig. 8. The first degradation step occurred between 478 and 700 °C, resulting in 35% weight loss, primarily attributed to the degradation of PEEK.^74^ Thus, sintering was done at 350 °C.^75^ The subsequent degradation steps occurred within the temperature range of 700–770 °C, which is accompanied by a weight loss of 6%.^76^ This weight loss corresponded to the degradation of organic compounds, CTAB, and unreacted nitrates. The low weight loss reflected the high thermal stability of the Cu-MBGNs, which limited the degradation process. As discussed in the FTIR section, the increased degradation temperature can be attributed to the weak interaction between PEEK and Cu-MBGNs. Notably, the degradation of titania was not observed up to 900 °C, confirming its high thermal stability.^77^ The char yield was ∼20%, indicating the percentage of non-degraded TiO_2_ and Cu-MBGNs.

The TGA result of bilayer composite coating deposited on 316L SS was in good agreement with previous findings reported in ref. 32, 66 and 78.

### 3.6. XRD analysis

**Fig. 9:**
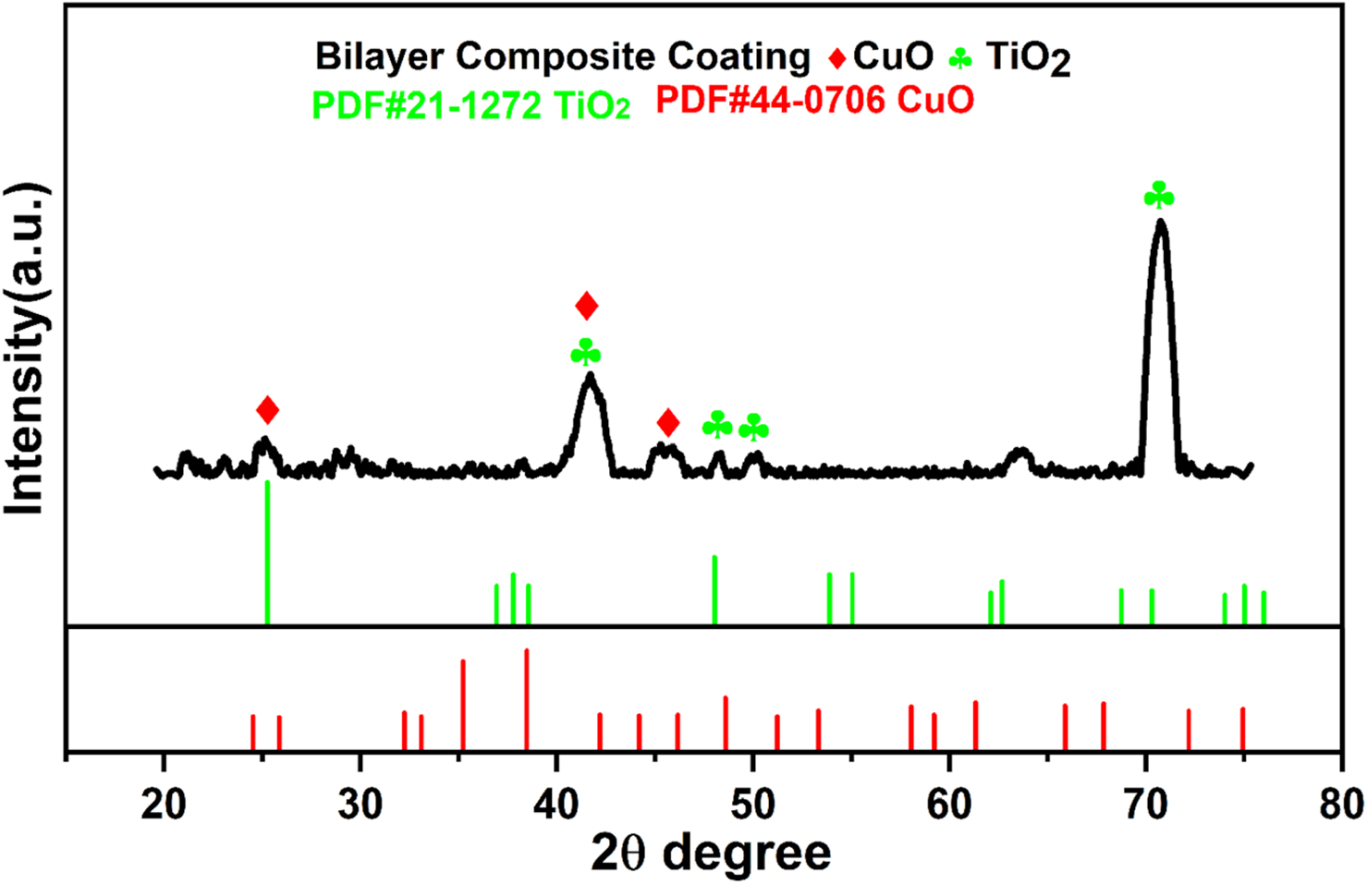
XRD spectrum of bilayer composite coating and relevant indexing and matching of the relevant peaks.

Fig. 9 shows the XRD pattern of the bilayer composite coating. The XRD pattern exhibited the semicrystalline nature of the coating. The peak at 2*θ* = 25.7° depicted the presence of CuO and moved towards the higher values of the Bragg angle, as reported elsewhere.^79,80^ This implies that after sintering, the lattice parameters are expanded and shifted from the angle to higher values. Furthermore, the peaks appeared at 2*θ* = 43.8°, 54.8°, and 75.1°, confirming the presence of TiO_2_ (anatase), and these data matched with JCPDS #21-1272. Similarly, findings have been reported in previous studies.^81,82^ The intensity of the diffraction peak of TiO_2_ increased after sintering, as shown by the XRD pattern, and the high value of impedance in the EIS spectra, as shown in Fig. 15.

The XRD results agree with the FTIR results, and both results show the presence of TiO_2_ and Cu-MBGNs in the bilayer composite coating deposited on 316L SS. PEEK is a semicrystalline material^83^ that serves as a matrix material, while TiO_2_/Cu-MBGNs are used as reinforcing agents in bilayer composite coatings. The XRD spectrum showed distinct peaks belonging to TiO_2_, and Cu-MBGNs confirmed the presence of these crystalline phases in the bilayer composite coating. However, these distinct peaks associated with TiO_2_ and Cu-MBGNs were relatively broad peak widths with lower intensities compared to fully crystalline material, suggesting the semicrystalline nature of bilayer composite coating.

### 3.7. Surface properties

Surface properties, such as surface roughness and wettability, are essential in the context of preventing biofilm formation and facilitating the attachment of osteoblast cells.^84^ The uncoated 316L SS substrate was used as the reference sample. Surface roughness is the most critical factor in implant surfaces due to its direct interaction between microbes and cell attachment. Furthermore, surface roughness affects the performance of an implant. The osteoblast cells favourably attach to the surface with an average roughness of 1–2 μm.^61,85^ This study measured the average surface roughness (*R*_a_) for bilayer composite coating as 1.20 ± 0.08 μm. The *R*_a_ value of the bilayer composite coating reported in this study is comparable with that reported in ref. 50 and 35. The *R*_a_ of bilayer composite coatings is less than that of PEEK coating deposited on 316L SS (the *R*_a_ value for PEEK was taken from our previous study^86^), which could be attributed to the difference in particle size and coating thickness.

Furthermore, the top layer may have infiltrated the bottom PEEK layer. The bilayer composite coating was subjected to sintering (as described in Subsection 2.2), which may have resulted in further compactness of the coating and infiltration of TiO_2_ and Cu-MBGNs in the pores of the PEEK layer. The relatively higher value of average roughness of the bilayer composite coating compared to that of the 316L SS can induce better adhesion of bone cells and related proteins. It can stimulate the formation of the extracellular matrix.^87^

The wettability of bilayer composite coating deposited on 316L SS implant is closely related to biological response in the human body.^88^ The critical protein attachment,^89^ cell adhesion,^90^ cell proliferation and behaviour^91^ to the surface can be adversely affected due to high hydrophobicity or hydrophilicity, which causes complications for bone tissue engineering. Menzies and Jones^87^ and Arias *et al.*^92^ reported that the ideal values of the contact angle for bone tissue application is between 35° and 80°. The measurement of the contact angles of 316L SS and bilayer composite coating are 90° ± 3° and 69° ± 2°, respectively. This value of the contact angle of bilayer composite coating is in good agreement with the accepted range of contact angles necessary for biological response.^92^ Furthermore, the contact angle value of the bilayer composite is slightly different from previously reported values of contact angles^31,52,92^ because of the difference in the composition of the bilayer composite coating. The moderately hydrophilic behavior of the bilayer composite coating is attributed to the TiO_2_ and Cu-MBGNs. The TiO_2_ is hydrophilic in nature,^93^ while the Cu-MBGNs contain silanol groups (Si–OH, which is formed due to the hydration of siloxane bond (Si–O–Si) on the surface of MBGNs), which are also hydrophilic. The synergistic effect of TiO_2_/Cu-MBGNs caused the contact angle of bilayer composite coating to decrease.

**Fig. 10:**
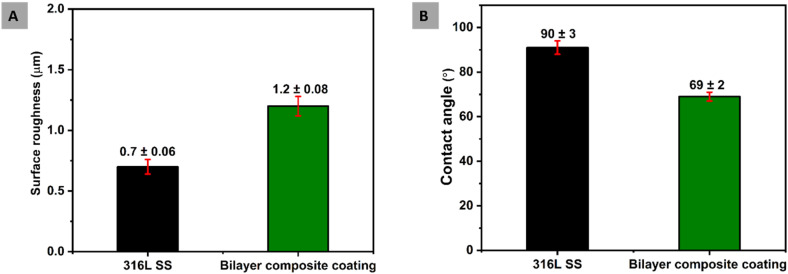
(A) Comparison of surface roughness of bilayer composite coating with 316L SS and (B) comparison between contact angles of bilayer composite coating and 316L SS.

Fig. 10 shows a comparison between the surface properties of the 316L SS and bilayer composite coating. Therefore, the bilayer composite coating exhibited moderately hydrophilic behavior, which can assist in the initial protein attachment and subsequent proliferation of bone-forming cells.^84^

The surface topography of bilayer composite coating can impact wettability. Thus, rougher surfaces would improve wettability due to the enhanced contact sites between the water and the surface. The hydrophilic nature of the coating can prevent protein denaturation. Thus, a contact angle of 69° ± 2° can be considered suitable for osseo-integration.

### 3.8. Bend test

**Fig. 11:**
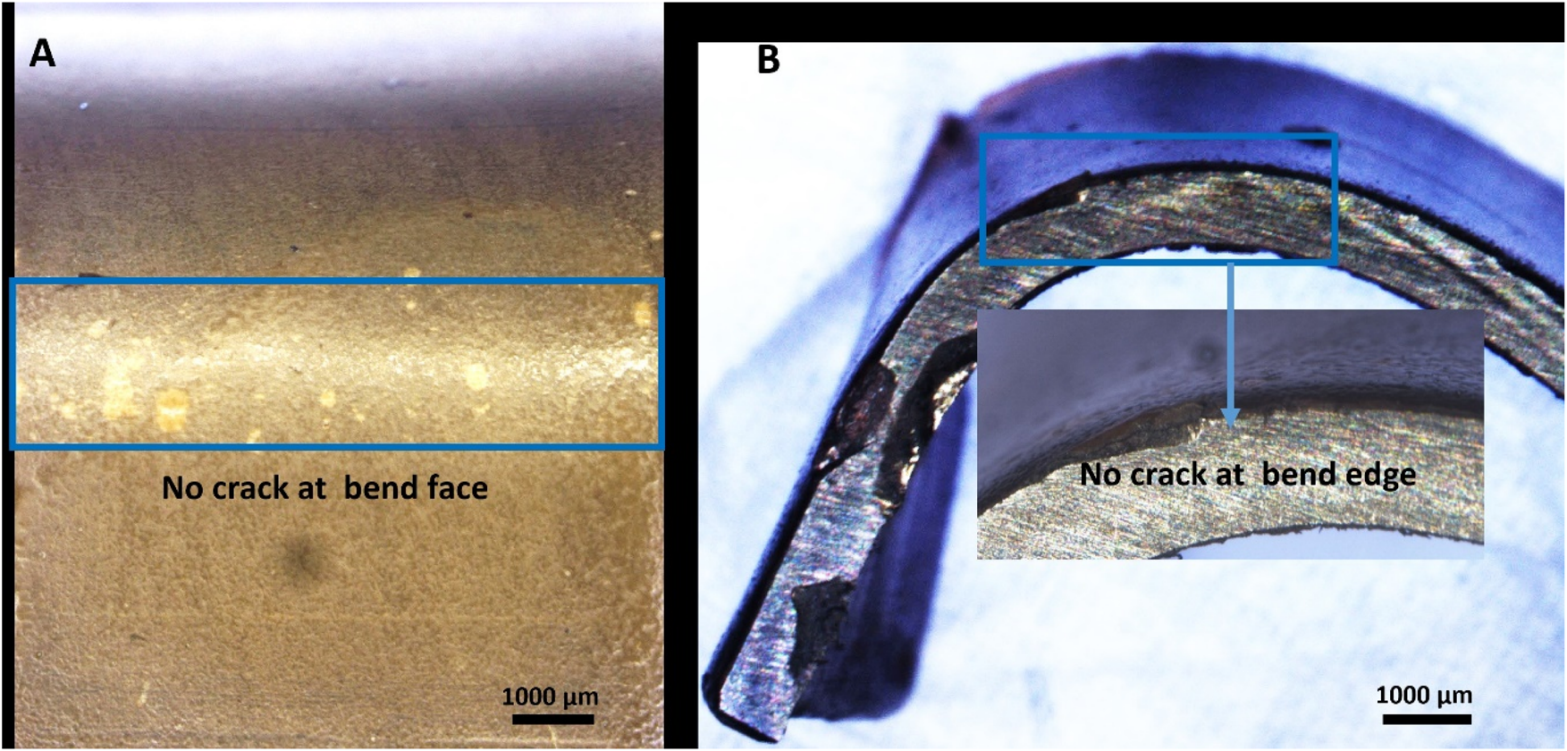
Stereomicroscope images showing the results of the bend test from different angles: (A) concave and (B) convex sides.

The adhesion strength of the bilayer composite coating deposited on the 316L SS substrate was determined by a bend test, as shown in Fig. 11. The bilayer composite-coated sample was manually bent at 180°, and stereomicroscope images showed no discernible cracks in the bilayer composite coating or delamination of the coating at the centre or edges of the bilayer composite coating (Fig. 11). The bending test result was evaluated according to ASTM standard A1122. The adhesion strength of the coating was rated as ‘5B’.^36^ The bending strength of the bilayer composite coating confirmed the excellent adhesion strength and resistance to deformation upon application of the bending load. The bilayer composite coating did not exhibit cracks at the site of the highest stress (marked by the blue box in Fig. 11A and B). Similar results for the PEEK-based composite coatings were reported in the literature.^36^ The excellent adhesion strength is attributed to the carefully selected sintering temperature of the PEEK layer as the first layer and the second layer of TiO_2_/Cu-MBGNs as reinforcing agents. The appropriate sintering temperature allowed the TiO_2_ and Cu-MBGN particles to interdiffusion in the PEEK layer. Thus, the bilayer composite coating provided a strengthening effect (resistance plastic deformation) upon applying the bending load.

The bilayer composite coating was neither delaminated nor cracked at the bend face and edge. The poor adhesion of the PEEK coating is attributed to the inert nature of PEEK, which restricts the bonding between the PEEK and TiO_2_/Cu-MBGN particles present in the coating. However, in the current study, the coatings exhibited excellent bending strength and adhesion strength. This could be attributed to the mesoporous nature of Cu-MBGNs and nano-sized TiO_2_ particles, which can create a rough interface with an oxide layer of 316L SS.^94^ Thus, it induced mechanical interlocking between the bilayer composite coating and 316L SS, which resulted in strong adhesion between bilayer composite coating and 316L SS substrates. Furthermore, previous studies on the PEEK-based composite coatings focused on the surface properties, biological properties, and release kinetics of specific drugs,^32,47,48,68,95^ but there is a limited study in which the adhesion strength of PEEK-based BG composite coating was evaluated through a bend test.^36^ The bilayer composite coating showed excellent adhesion strength through the bend test than previously reported in a study.^36^

### 3.9. Antibacterial study

**Fig. 12:**
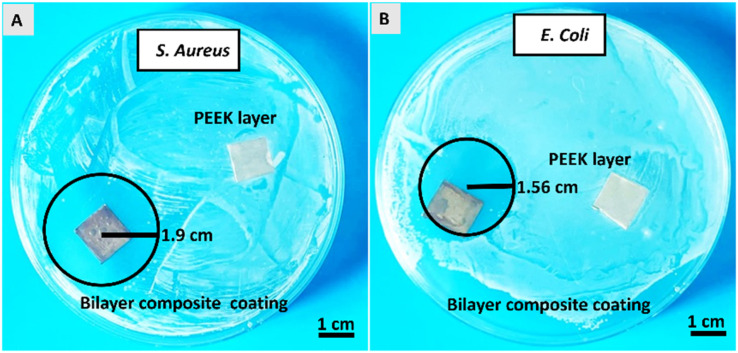
Antibacterial activity of bilayer composite coating against (A) *S. aureus* and (B) *E. coli* strains.

**Fig. 13:**
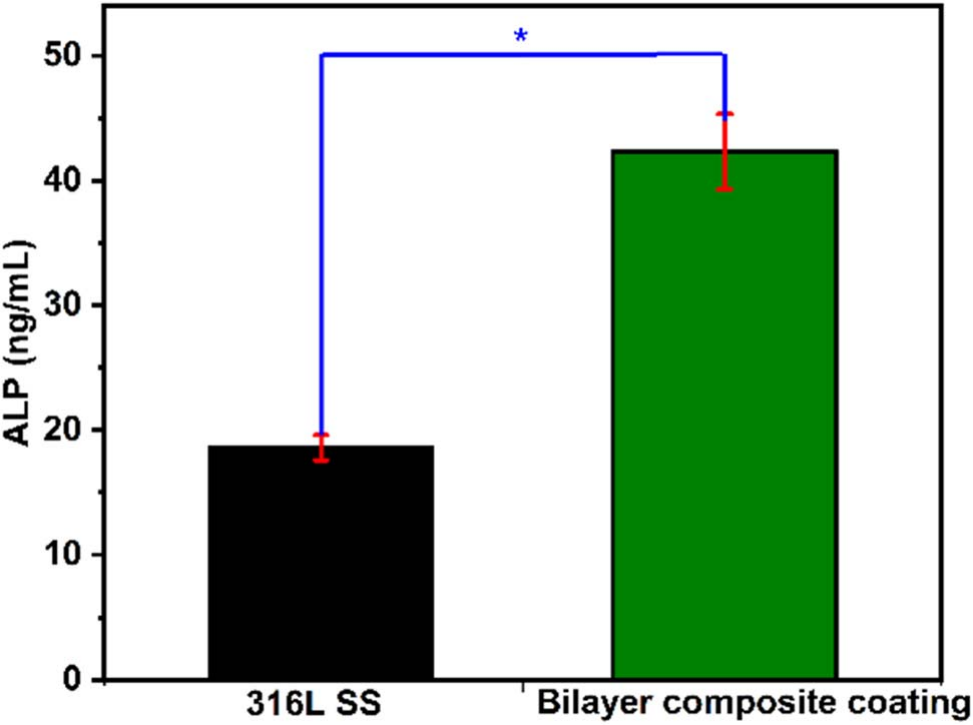
ALP release (ng mL^−1^) from 316L SS and bilayer composite coated 316L SS, where * shows that the difference between 316L SS and bilayer composite coating is statistically significant at *p* < 0.05 (data represent ± standard deviation of three samples).

The antibacterial activity of the PEEK coating and Cu-MBGNs loaded with the coatings was evaluated against *S. aureus* and *E. coli* strains. The PEEK coating was used as a control sample. Images of Petri dishes containing inoculated agar and coatings after 24 h were taken in a blue background to enhance the visibility of zones of inhibition displayed by bilayer composite coating.^31,96,97^Fig. 13 shows that the bilayer composite coating exhibited inhibition zones with a radius of 1.9 ± 0.14 cm and 1.56 ± 0.09 cm against (Fig. 12A) *S. aureus* and (Fig. 12B) *E. coli*, respectively.

The release of Cu^2+^ ions from bilayer composite coatings is responsible for introducing antimicrobial activity. The *S. aureus* contains a single lipid bilayer of peptidoglycan (20–80 nm), which becomes attractive to positive charges (as it has negatively charged surface polymers, such as teichuronic and lipoteichoic acids). Thus, the zone of inhibition depended on the action of release of metallic ions, which diffused quickly in the microbial cells due to attraction. This resulted in cell apoptosis through cell membrane disruption and the denaturation of protein.^52^*E. coli* is less attractive towards metallic ions as it has a highly organized compact structure, which acts as a permeability barrier. Ultimately, diffusion is prevented. Thus, the zone of inhibition appeared to be smaller against *E. coli*. Additionally, Cu^2+^ tolerance mechanisms in *E. coli*, such as copper removal by metallothioneins and activation transmembrane Cu^2+^ export occurring from the cytoplasm into the periplasmic space or the extracellular matrix, can further explain why the inhibition zone was smaller against *E. coli* than *S. aureus*.^24^ Furthermore, the bilayer composite coating demonstrated an improved antibacterial effect against *S. aureus* and *E. coli* compared to previous studies conducted on PEEK-based bioactive glass doped with different metallic ions, such as Ag, Mn, lawsone, curcumin (natural herbs), and chitosan incorporated in composite coating.^31,32,35,49,98^

### 3.10. Cellular study

To understand the bone formation and mineralization processes in the coated and uncoated samples, the release of a key protein (ALP) in the bone matrix was assessed. The ALP release was significantly higher for the bilayer composite-coated 316L SS than for the 316L SS (as shown in Fig. 13). This may be attributed to the release of bioactive ions and the improved surface topography of 316L SS.^99^ It is assumed that the Cu-MBGNs incorporated into the bilayer composite coating may have released Cu^2+^ ions to induce osteogenic activity and ultimately enhance ALP release.^100^ Cu^2+^ ions were reported to enhance ALP release by the activation of signalling pathways, such as the Wnt/β-catenin pathway and the mitogen-activated protein kinase (MAPK) pathway.^101,102^

However, the improved surface topography may also influence cell adhesion and differentiation. Cu^2+^ ions can also inhibit the activity of osteoclast cells by the induction of apoptosis, inhibition of enzymes involved in osteoclast activity (such as cathepsin K), or disruption of the nuclear factor-kappa B (NF-κB) signalling pathway, which is critical for osteoclast formation and function.^103,104^

### 3.11. Tribological study

Tribological properties of PEEK coating (thickness ∼66 μm)^40^ and bilayer composite coating (thickness ∼42 μm) were investigated by a pin-on-disc test conducted under DMEM-lubricated conditions. The tribological properties of bilayer composite coating were compared with PEEK coating (the tribological properties of PEEK coating deposited on 316L SS are described in our previous study^40^). The effect of the TiO_2_/Cu-MBGN layer infused into the PEEK layer was studied regarding tribological properties.

The PEEK coating with a thickness of ∼66 μm (ref. 40) and bilayer composite coatings were subjected to tribological study at 10 N under DMEM lubricating condition (which is more relevant to physiological conditions). The PEEK coating in the presence of DMEM exhibited instability due to delamination. Thus, the functional integrity of the PEEK coating under physiological conditions cannot be established due to delamination and negative CoF. However, the bilayer composite coating exhibited stable behaviour throughout the sliding distance. The CoF of the bilayer composite coating remained constant at ∼0.032 in the presence of DMEM throughout the sliding distance. CoF behaviour confirmed that bilayer composite coating is wear-resistant and, thus, suitable for orthopedic applications.

**Fig. 14:**
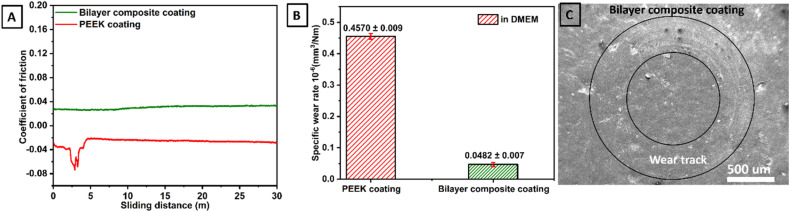
(A) CoF measured as a function of sliding distance for both PEEK and bilayer composite coatings under wet conditions, *i.e.*, in the presence of DMEM, (B) comparison between specific wear rates and (C) SEM image of wear track of bilayer composite coating.

The CoF of bilayer composite coating decreased under DMEM lubricating conditions compared to that of the CoF of bilayer composite coating under dry sliding conditions. The CoF of bilayer composite coating was reduced under DMEM lubricating conditions due to decreased friction between bilayer composite coating and counter surface.^105^ There is a possibility that DMEM molecules can migrate to the bilayer composite coating surface and form a thin layer that acts as a lubricant. DMEM molecules can also penetrate minor grooves and scratches on bilayer composite coating, which reduces the roughness of the surface and leads to a decrease in the CoF of bilayer composite coating. Furthermore, DMEM solution can wet the surface of bilayer composite coating, producing a thin film of lubricant, thus reducing the friction between bilayer composite coating and counter surface. The results of tribological tests of PEEK and bilayer composite coatings in DMEM are expressed in terms of CoF *vs.* sliding distance (Fig. 14A). Fig. 14B shows a comparison between the specific wear rates, and Fig. 14C shows the SEM image of the wear track.

The specific wear rates of PEEK and bilayer composite coatings were also determined under DMEM lubricating conditions. In DMEM, the bilayer composite coating (Fig. 14B) showed better results than the PEEK coating. The specific wear rate of bilayer composite coating was (0.0482 ± 0.007) × 10^−6^ mm^3^ Nm^−1^, while PEEK coating showed a particular rate of wear of (0.4570 ± 0.009) × 10^−6^ mm^3^ Nm^−1^, indicating that PEEK coating delaminated from 316L SS in the presence of DMEM (Table 2). It can be assumed that the DMEM is a cell culture media that contains high concentrations of various ions (Na^+^, K^+^, Ca^2+^, Mg^+^, Cl^−^ and PO_4_^3−^) and solutes (amino acids, glucose, salts, vitamins, hormones, and growth factors). Owing to the semi-crystalline nature of PEEK, it may be susceptible to hydrolytic degradation.^106^ This may lead to the breakdown of the polymer chain, which results in the formation of cracks and fissures in the PEEK coating during tribological tests. These cracks and crevices can provide pathways for DMEM to penetrate the coating. This penetrated DMEM can attack the underlying 316L SS and lead to the delamination of the PEEK coating from 316L SS.^40,107^ In the tribology test, sliding contact could lead to wear mechanisms, such as abrasion and adhesion, which gradually delaminated the PEEK coating in the presence of DMEM.

**Table 2:** Tribological performance of PEEK and bilayer composite coatings in DMEM under a 10 N applied normal load

Condition	Coating	Applied	Accumulative wear	Specific wear rate	Average
		Load (N)	Volume (mm^3^)	(×10^−6^ mm^3^ N^−1^ m^−1^)	CoF
DMEM	PEEK coating	10	137.10 ± 0.06	0.4570 ± 0.009	0.0758
	Bilayer composite coating	10	14.46 ± 0.02	0.0482 ± 0.007	0.0302

Fig. 14C shows the SEM image of the wear track belonging to bilayer composite coating where the coating did not delaminate after pin on disc test conducted under 10 N applied normal load in the presence of DMEM. In the bilayer composite coating, TiO_2_ and Cu-MBGNs in the top layer reinforced the PEEK layer upon sintering. This improves the mechanical properties such as resistance to delamination and wear under dry and DMEM lubricating conditions. The compact TiO_2_/Cu-MBGNs layer infused in the PEEK layer acts as a barrier between the DMEM and 316L SS. Thus, DMEM could not penetrate the underlying 316L SS, which further reduced the risk of delamination of the bilayer composite coating. Table 2 illustrates the performance of PEEK and bilayer composite coatings under wet conditions.

This strong adhesion strength of the bilayer composite coating may be attributed to the following mechanisms. After SEM analysis of the bilayer composite coating at the surface, the morphology and topography showed that TiO_2_/Cu-MBGNs were uniformly distributed, and clusters of these particles were observed at specific sites. Furthermore, porosity or micro voids were not present in the bilayer composite coating. Similarly, the cross-section of bilayer composite coating, with a thickness of ∼42 μm, was also mapped to observe the diffusion of TiO_2_/Cu-MBGNs in the PEEK layer after sintering at 350 °C. It can be assumed that even the PEEK might be decomposed at sintering temperature, while the residual carbon might form a strong bond with a protective oxide layer of 316L SS. The TiO_2_/Cu-MBGNs present on the sintered PEEK layer might react with this residual carbon. Furthermore, the sintering of the TiO_2_/Cu-MBGNs layer deposited on the sintered PEEK layer resulted in melting and fusion at the interface between PEEK particles and TiO_2_/Cu-MBGNs because the sintering created a porous and rough surface on the PEEK layer, where these TiO_2_/Cu-MBGNs penetrated pores of the PEEK layer. The TiO_2_/Cu-MBGNs were embedded in a PEEK layer and acted as reinforcing materials in the bilayer composite coating, resulting in a mechanically interlocked structure between the PEEK layer and TiO_2_/Cu-MBGNs layer, which prevented the delamination of the bilayer composite coating by anchoring these TiO_2_/Cu-MBGNs firmly in the PEEK matrix. Thus, it was concluded that the surface largely consisted of PEEK with particles of TiO_2_/Cu-MBGNs embedded in the bilayer composite coating system (which supports the proposed strengthening mechanism in the current study).^108^

### 3.12. Electrochemical studies

**Fig. 15:**
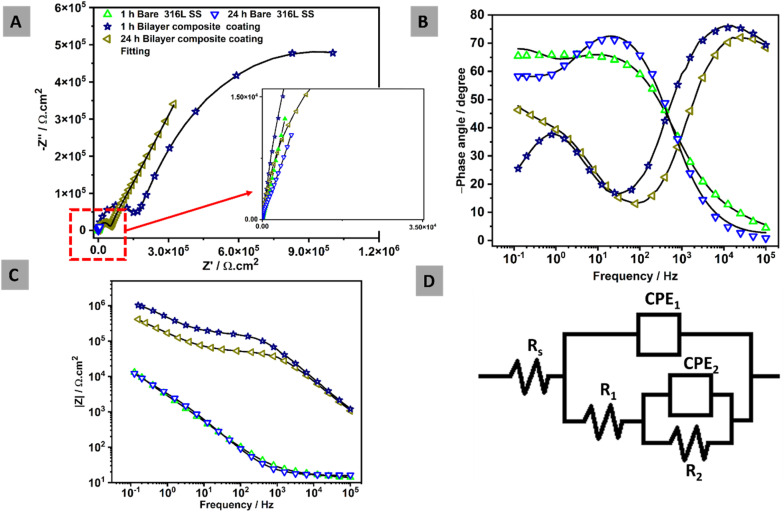
EIS spectra over immersion time in PBS at 37 ± 1 °C: (A) Nyquist plots, (B) Bode phase angles, (C) Bode impedances, and (D) equivalent electric circuit.

EIS measurements were conducted of all bare 316L SS and bilayer composite coating after 1 h and 24 h of immersion in PBS at 37 ± 1 °C, as shown in Fig. 15. The EIS data were analyzed, and the results were displayed in Nyquist and Bode plots. The EIS data were also subjected to fitting in ZSimpWin software with an appropriate electrical equivalent circuit to determine the values of the relevant parameters. Nyquist plot, which is a graphical representation of electrical behavior in terms of impedance, was plotted between real impedance at the *x*-axis and imaginary impedance at the *y*-axis.

Nyquist plots of all samples analyzed after 1 h and 24 h in PBS, as shown in Fig. 15A. The semi-capacitive arcs in the Nyquist plot of bare 316L SS remained unchanged over the immersion time, indicating that no corrosion was observed. In contrast, the bilayer composite coating over immersion time depicted a higher semi-capacitive arc,^40^ indicating higher corrosion resistance. Fig. 15B shows that the Bode phase plots with two-time constants are observed in all samples.

For the 316L SS substrate, the humps observed at the lowest frequencies (around 1 Hz) in the Bode-phase diagram (Fig. 15B) after 1 and 24 h are attributed to an inner compact layer of passive film early formed on the steel substrate, which acts as a strong diffusion barrier. However, the humps at higher frequencies are related to the outer layer of the oxide passive film on the 316L SS. The bilayer composite coating demonstrated a high-frequency hump at ∼10 kHz (at both 1 and 24 h immersion) due to the strong physical barrier property of the bilayer composite coating. The low-frequency response at around 1 Hz is again attributed to the inner layer of the passive film early formed on the steel substrate.

Fig. 15C presents the Bode impedance plots, where the low-frequency impedance value at 10^−1^ Hz (|*Z*| 0.1 Hz) is a common indicator for observing the properties of the coating. All curves illustrated comparable results except for the bilayer composite coating, which implies that no corrosion was observed over the immersion time of 24 h in PBS. The bilayer composite coatings showed higher impedance values over the immersion times (1 and 24 h) compared to 316L SS. The main reason for this is associated with the strengthening effect of TiO_2_/Cu-MBGNs in the bilayer composite coating. The EIS measurements were fitted by an electric equivalent circuit in Fig. 15D to determine the electrochemical behavior of the samples. Fig. 15D shows two-time constants, which are used to study the response of bare 316L SS and bilayer composite coating. In the electrical equivalent circuit, *R*_s_ represents the solution resistance. Whereas, CPE_1_ and *R*_1_ represented the capacitance of the double layer and resistance of the outer surface of bilayer composite coating and oxide layer of 316L SS, respectively. CPE_2_ and *R*_2_ represent the capacitance and resistance of the inner layer of bilayer composite coating and inner passive oxide layer of 316L SS, respectively.

Generally, CPE is used instead of pure capacitance to define the non-ideal capacitive behavior of the element, which is mainly attributed to the uneven growth of the generated layer.^109^ Fit values were measured for each sample, and fitting showed a Chi-square value in the 10^−4^ range, which is an indication of very good fits. The equivalent capacitance values of different elements in Fig. 15D can be calculated using CPE values (CPE_1_, CPE_2_, *n*_1_, and *n*_2_) with their respective resistance according to eqn (11),^81^ where *Q*, *R*, and *n* represent the charge, resistance, and exponent of the constant phase element for porous and passive barrier coating, respectively:

11 CPE = *Q*^1/*n*^*R*^(1−*n*)/*n*^

Table 3 EIS fitting values for each element in the electrical equivalent circuit.

**Table 3:** EIS circuit fitting for different samples in PBS at 37 ± 1 *°*C after (1 h and 24 h) of immersion time

Samples	*R*_s_ (Ω cm^2^)	CPE_1_ (μΩ^−1^ s^*n*^ cm^2^)	*n*_1_ -	*R*_1_ (Ω cm^2^)	CPE_2_ (μΩ^−1^ s^*n*^ cm^2^)	*n*_2_ -	*R*_2_ (Ω cm^2^)	*χ* ^2^ fits
1 h Bare SS	1.32	68.7	0.7	5.52 × 10^3^	22.1	0.8	6.04 × 10^3^	2.53 × 10^−4^
24 h Bare SS	1.62	49.6	0.8	8.81 × 10^3^	40.6	0.8	7.22 × 10^3^	4.05 × 10^−4^
1 h Bilayer composite coating	1.74	0.06	0.7	1.56 × 10^7^	0.006	0.8	1.5 × 10^5^	2.62 × 10^−4^
24 h Bilayer composite coating	1.17	0.007	0.8	4.56 × 10^5^	2.5	0.8	9.07 × 10^4^	2.73 × 10^−4^

The bilayer composite coating demonstrated an impedance value of ∼1.56 × 10^7^ Ω cm^2^ after 1 h and decreased to ∼4.56 × 10^5^ Ω cm^2^ after 24 h. However, the impedance value of the bilayer composite coating was greater than that of the bare 316 L SS (8.81 × 10^3^ Ω cm^2^) even after 24 h immersion in PBS. Furthermore, the impedance value of the bilayer composite coating decreased after 24 h of immersion in PBS, but it was significantly higher than the impedance values of the bare 316L SS. This indicates that the bilayer composite coating showed improved corrosion resistance owing to its higher impedance of ∼4.56 × 10^5^ Ω cm^2^ after 24 h compared to bare 316L SS in PBS, as shown in Table 3.

Overall, the bilayer composite coating developed in this study exhibited suitable physical, mechanical, and electrochemical properties. However, detailed *in vitro* bioactivity studies, antibacterial studies (quantitative), and *in vitro* cell culture studies using direct and indirect methods should be carried out to confirm the suitability of the developed coatings for further *in vivo* and eventually clinical applications. Nevertheless, the significant improvement in corrosion and wear resistance is attributed to this study and is a significant milestone for PEEK-based composite coatings for orthopaedic applications.

## 4. Conclusions

The present study investigated the synergistic effects of TiO_2_/Cu-MBGNs in the bilayer composite coating deposited on 316L SS *via* EPD. First, a layer of PEEK was deposited on 316L SS substrate, followed by sintering at 350 °C for 30 min. Then, a second layer of TiO_2_/Cu-MBGNs was deposited and sintered again. PEEK layer and bilayer composite coating were analyzed through SEM/EDS and FTIR analyses. Moreover, XRD results demonstrated that TiO_2_/Cu-MBGNs are found in bilayer composite coating. The bilayer composite coating exhibited excellent mechanical strength, as confirmed by the bend test. The bilayer composite coating showed potential antibacterial activity against *S. aureus* and *E. coli*. A tribological study confirmed that the bilayer composite coating bears a 10 N load under DMEM, which mimics the human body environment. The EIS results of the bilayer composite coating in PBS showed good corrosion resistance, which is attributed to the synergistic effect of TiO_2_ and Cu-MBGNs with an increase in the barrier properties compared to the PEEK coating and the 316L SS.

## Data availability

The data that support the findings of this study are available from the corresponding author, Prof. Muhammad Atiq Ur Rehman (atique1.1@hotmail.com), upon request.

## Conflicts of interest

There are no conflicts to declare.
